# Selective detection of dopamine with an all PEDOT:PSS Organic Electrochemical Transistor

**DOI:** 10.1038/srep35419

**Published:** 2016-10-14

**Authors:** Isacco Gualandi, Domenica Tonelli, Federica Mariani, Erika Scavetta, Marco Marzocchi, Beatrice Fraboni

**Affiliations:** 1 Dipartimento di Chimica Industriale ‘Toso Montanari’, Università di Bologna, Viale Risorgimento 4, 40136 Bologna, Italy; 2 Dipartimento di Fisica e Astronomia, Università di Bologna, Viale Berti Pichat 6/2, 40127 Bologna, Italy

## Abstract

An all PEDOT:PSS Organic Electrochemical Transistor (OECT) has been developed and used for the selective detection of dopamine (DA) in the presence of interfering compounds (ascorbic acid, AA and uric acid, UA). The selective response has been implemented using a potentiodynamic approach, by varying the operating gate voltage and the scan rate. The trans-conductance curves allow to obtain a linear calibration plot for AA, UA and DA and to separate the redox waves associated to each compound; for this purpose, the scan rate is an important parameter to achieve a good resolution. The sensitivities and limits of detection obtained with the OECT have been compared with those obtained by potential step amperometric techniques (cyclic voltammetry and differential pulse voltammetry), employing a PEDOT:PSS working electrode: our results prove that the all-PEDOT:PSS OECT sensitivities and limits of detection are comparable or even better than those obtained by DPV, a technique that employs a sophisticate potential wave and read-out system in order to maximize the performance of electrochemical sensors and that can hardly be considered a viable readout method in practical applications.

Electrochemical methods have been applied for the detection of many analytes in a great variety of matrices, the most widespread being the common blood glucose detection systems used by diabetic patients. Electrochemical sensors have attracted much attention thanks to major advantages such as short analysis times, when compared to spectroscopic techniques, and simple experimental procedures which can be applied to a variety of physiological samples^1^,^2^. They mostly operate in amperometric mode and thus require the use of a reference electrode.

The selective detection of dopamine (DA), one of the most significant neurotransmitters in biological organisms^3^, is currently a subject of significant interest. The rapid and accurate determination of DA is of great importance in the diagnosis of neurological disorders, such as Parkinson’s disease, autism, schizophrenia^4^. The main problem related to the electrochemical detection of dopamine is that its oxidation potential is close to that of other endogenous substances such as uric acid (UA) and ascorbic acid (AA), which leads to poor selectivity and sensitivity in DA detection^5^.

To overcome these problems, different kinds of electrochemical sensors using amperometric transduction have been proposed in the literature^6^,^7^,^8^. The use of a conductive polymer as electrode coating allowed to selectively detect AA, UA and DA, and interesting results in terms of sensitivity and peak resolution have been obtained using a sensitive technique such as differential pulse voltammetry (DPV)^9^. Nevertheless, dopamine concentration can be very low in biological samples (for blood <1 nmol L^−1^) and such sensors do not exhibit an adequate sensitivity for real-life applications.

A very interesting device that has recently attracted large attention for its high sensitivity in bio analyte detection is the organic electrochemical transistor (OECT). An OECT operates at low voltages (less than 1 V) and it is very easy to fabricate, being composed by a stripe of conductive polymer that works as a channel, and by another electrode, usually a metal, that acts as a gate^10^,^11^. Furthermore, it does not require any reference electrode and the signal can be rapidly collected with a very simple and low cost readout electronics. OECTs can work as chemical sensors when the analytes affect the electrochemical processes that control the doping of conductive polymers and thus change the current that flows in the channel. The transistor configuration guarantees signal amplification since small potential changes due to the analyte presence are followed by a large variation of the channel current, thus assuring high sensitivity and a very low limit of detection (LOD) of the analytical detection.

Conducting polymers have been extensively studied as electrode coatings, to enhance the physico-chemical properties of bare electrodes^12^. They are effective when used as amperometric sensors since they exhibit anti-fouling properties and can behave as redox-mediators toward several analytes, significantly enhancing sensitivity and selectivity of the analytical detection, and even lowering the LOD value. In the last years, poly(3,4 ethylenedioxythiophene) (PEDOT) is receiving great attention in the development of modified electrodes, due to its low-energy band gap^13^ which makes it suitable for electro-optical applications. Moreover, bearing the ethylenedioxy substituent in the 3 and 4 positions of the polythiophenic ring, it is regiochemically better defined than unsubstituted polythiophene.

The counter anion incorporated into the polymer to compensate the positively charged PEDOT plays a key role in defining PEDOT properties: when the polystyrene sulphonate (PSS) polyanion is used the PEDOT:PSS system has proven to produce films and micro-structured systems with facile electrochemistry, high-ionic conductivities, good electrochemical stability and a capacitance suitable for practical use in electrochemical supercapacitors^14^,^15^,^16^,^17^,^18^. Moreover, thanks to its favorable features, PEDOT:PSS has been recently employed for the fabrication of all-plastic OECT^19^,^20^,^21^.

In an all-plastic OECT the metal gate electrode is replaced by PEDOT:PSS, significantly further reducing the fabrication cost and enabling the whole device preparation with a single-run, low cost deposition technique. Recently we demonstrated that an all PEDOT:PSS OECT can be used as sensor for AA, exploiting the capability of PEDOT:PSS itself to directly electrocatalyze AA oxidation, thus obtaining an enzyme-free, all-PEDOT:PSS chemical sensor^22^. The device operation mode is based on the extraction of charge carriers from the transistor channel, following AA oxidation at the gate electrode, which leads to a decrease of the drain current. The variation in hole concentration can be monitored by the measurement of the electrochemical potential with respect to a reference electrode, indicating that the OECT is ruled by the same electrochemical processes that rule the operation of chemically modified electrodes. It is evident that a drawback of such a device is its poor selectivity: any analyte able to undergo oxidation at the PEDOT:PSS gate electrode can contribute to the experimental response and thus the sensor selectivity is a key issue to address.

In this work we demonstrate that all-PEDOT:PSS OECTs satisfy both the requirements of selectivity and of detection of low amounts of bioanalytes, thanks to their intrinsic signal amplification and to the lack of need for a reference electrode. The selective response for DA, UA and AA has been realized using a potentiodynamic approach (e.g. by identifying the operating gate bias voltage and the scan rate), opening new ways in the development of flexible, all-plastic devices for real-life applications. It is worth remembering that DA detection by OECTs with a metal gate electrode has already been described by other research groups. The first manuscript dealing with this topic was by H. Tang *et al.*, who investigated the use of OECTs having different gate electrodes (Pt, Au, graphite) and compared their responses to DA in terms of sensitivity and limit of detection. The OECT operation depends on both gate electrode and applied voltage and the device with a Pt gate electrode shows the highest sensitivity and a detection limit lower than 5 nM^23^. However, this first OECT sensor does not address the problem of the interferents in DA detection.

C. Liao *et al.* exploited a Pt gate electrode coated by Nafion or chitosan to overcome the issue of AA and UA interference; moreover, the sensor response was also enhanced with graphene flakes^24^. The devices with a metal gate modified with Nafion–graphene showed a detection limit down to 5 nM and an excellent selectivity in DA determination.

The novelty of our work arises both in the replacement of the metal electrode by PEDOT:PSS and in the use of transconductance measurements to discriminate among different analytes. We propose for the first time an all-PEDOT OECT sensor for DA detection, capable to handle interference with AA and UA without the need of introducing any additional modifying agent.

## Results and Discussion

**Figure 1:**
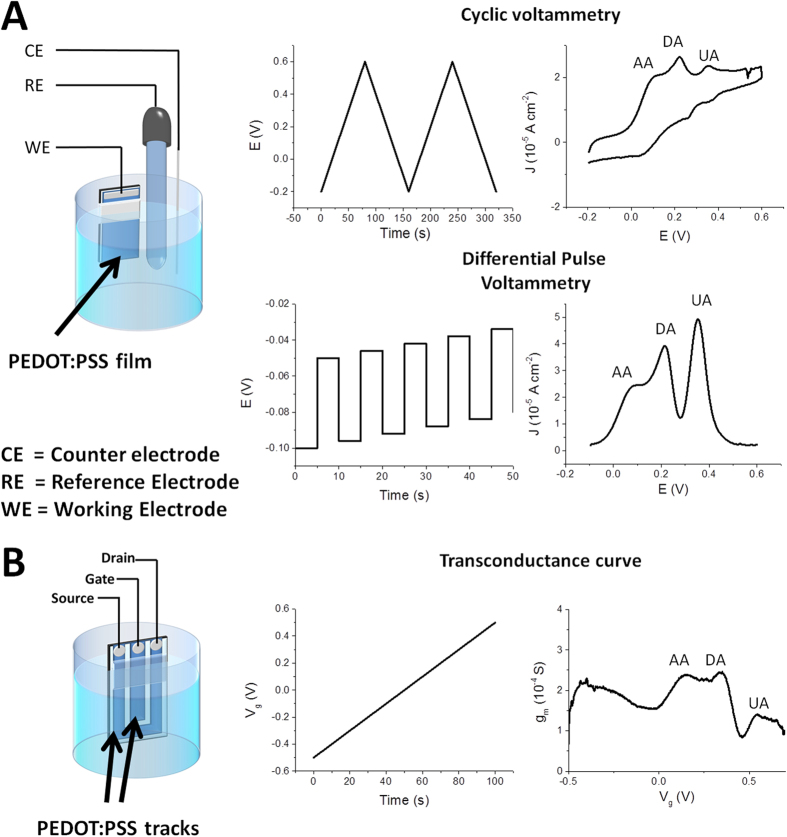
Experimental set up, potentials waves and responses for electrochemical experiments in the 3-electrode amperometric cell (**A**) and OECT configuration (**B**), respectively.

Figure 1 and Fig. SI1 show respectively the experimental set-ups and the working electrodes used for the electrochemical measurements in the 3-electrode amperometric cell and in the OECT configuration. Having already demonstrated^22^ that the all PEDOT:PSS OECT response is ruled by the analyte oxidation at the gate electrode, we studied the AA, DA, and UA responses at a PEDOT:PSS electrode, made exactly as an OECT gate electrode, in a classical three electrode cell by cyclic voltammetry (CV) and differential pulse voltammetry (DPV) at different scan rates.

**Figure 2:**
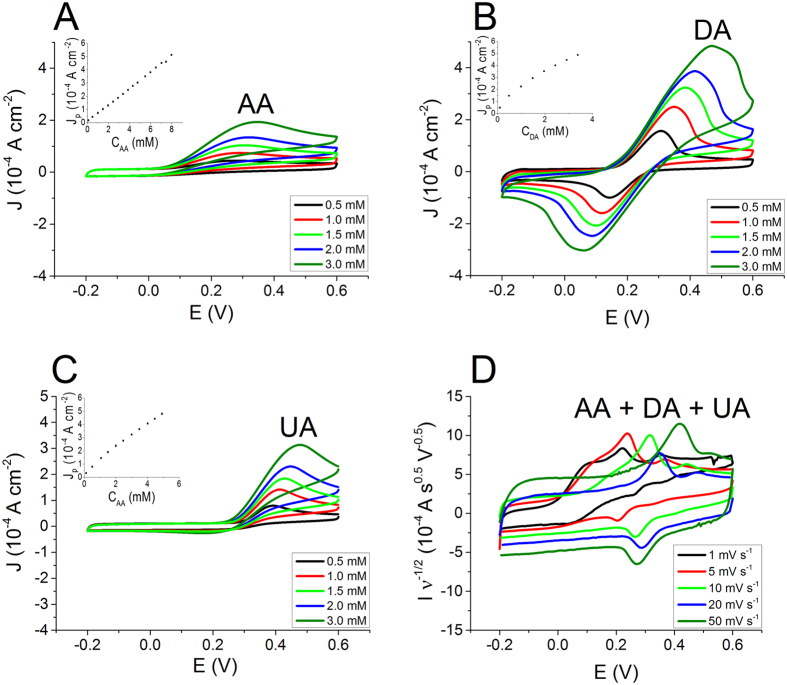
CV curves at 0.020 V s^−1^ recorded in a solution containing AA (**A**), DA (**B**) and UA (**C**) at different concentrations (insets: calibration plots). (**D**) CV curves recorded at different scan rates in a solution containing AA (1 mM), DA (1 mM) and UA (1 mM).

First of all, we assessed the CV electrochemical behavior of the three individual redox compounds. In a CV experiment the working electrode potential is ramped linearly versus time and the ramps in potential may be repeated as many times as desired (Fig. 1A). The current is measured at the working electrode and it is plotted versus the applied voltage (i.e., the working electrode’s potential) to give the CV trace. Figure 2 reports the CVs recorded for the three different analytes at different concentrations. The CV of each biomolecule exhibits a peak which is associated to the analyte electro-oxidation that occurs at a characteristic potential (V) measured with respect to a saturated calomel electrode (SCE). The peak potential value depends both on the thermodynamics and on the kinetics of the redox reaction. The peak current is linearly related to the analyte concentration (Fig. 2) and the slope is the sensitivity of the CV detection. Table 1 reports the anodic peak potential and the sensitivity of calibration line obtained by CV experiments at 0.020 V s^−1^ for each analyte. The sensor exhibits the best sensitivity for the DA detection, while AA shows the lowest redox signal.

Since the electrochemical processes under investigation occur at different potentials, it is possible to selectively determine the redox signal associated to each analyte when DA, AA and UA are simultaneously present in the solution and, consequently, to exploit it in order to determine its concentration. Figure 2D shows the CVs recorded at different scan rates in a solution containing AA (1.0 mM), DA (1.0 mM) and UA (1.0 mM). The graph points out that the scan rate affects the kinetics of charge transfer and, consequently, this parameter can be useful to control the intensities and the potentials of the redox waves. For each tested scan rate the UA peak is well separated from the AA and DA signals. The redox waves of DA and AA are not well-defined at the highest scan rates (0.010–0.050 V s^−1^), the best resolution being obtained at 0.001 V s^−1^. Such phenomena can be explained considering the slow kinetics of AA electro-oxidation: when the scan rate is lowered, the reaction has more time to occur and, consequently, a lower over-potential is required for the AA electro-oxidation thus leading to a separation from the DA peak, which occurs at a slightly more positive potential.

**Figure 3:**
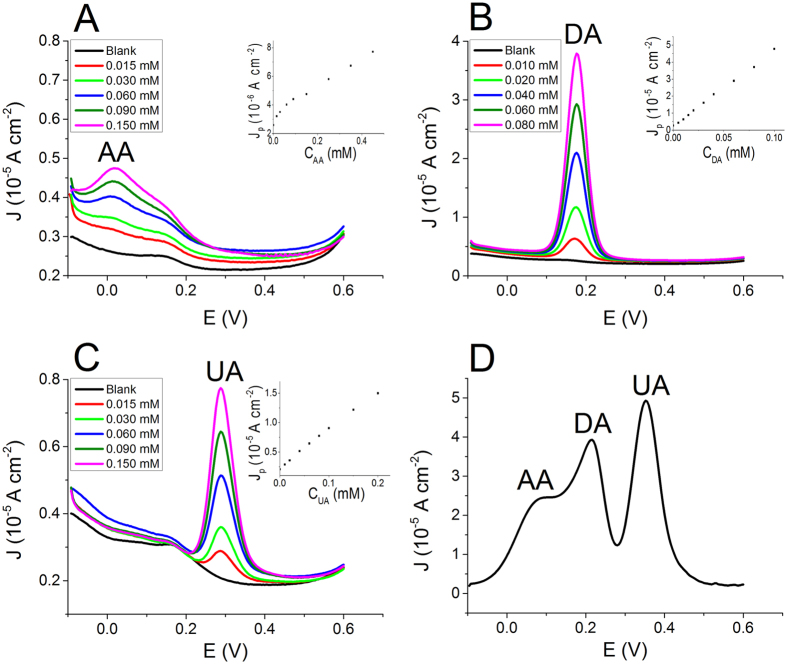
DPV curves recorded in a solution containing AA (**A**), DA (**B**) and UA (**C**) at different concentrations (Insets: calibration plots). (**D**) DPV curves recorded in a solution containing AA (1 mM), DA (0.07 mM) and UA (0.4 mM)

The electrochemical responses of the three bio-compounds were also studied by using DPV, a sophisticated electrochemical technique. During a DPV the working electrode is polarized with a series of regular voltage pulses superimposed on stair-steps (Fig. 1A). The current is measured immediately before each potential change and the difference between the currents measured after and before the pulse is used as signal. Such a procedure allows to minimize the capacitive current contribution due to the charging of PEDOT:PSS through the hole injection/extraction and the ions exchange with the solution. As a consequence, the measurement of the faradic current associated to the analyte reaction is more accurate than in a CV experiment and an higher sensitivity can be obtained. Figure 3 reports the DPVs recorded for solutions containing solely AA, DA or UA at different concentrations. The set-up parameters were chosen on the basis of the CV results that enabled the best separation between the peaks, i.e. a very low scan rate. Also in DPV each analyte exhibits a redox wave at a characteristic potential and the peak current linearly depends on the analyte concentration. Table 1 reports the parameters of the calibration plots obtained by DPV (Fig. 3) and also the DPV sensitivities normalized to the pulse amplitude of the potential wave in order to compare the DPV performance with the one of OECT sensors. The sensitivities must be expressed per voltage unit, because the pulse amplitude plays a key role in determining the intensity of faradic currents. It is worth noting that DPV exhibits, for each compound, a LOD value that is about one order of magnitude lower than that obtained by CV.

Figure 3D shows a typical DPV signal recorded for a solution containing 1 mM AA, 0.07 mM DA and 0.4 mM UA. The redox peak of each bio-analyte is clearly visible in the response and the resolution between the peaks is much better than the one obtained by CV. These results clearly show that the amperometric detection of AA, DA and UA can be fulfilled by using a PEDOT:PSS coated electrode by either CV or DPV. It is worth underlining that the performance of an amperometric sensor can be improved by using a potential wave that allows the minimization of the background capacitive current. This is the reason why DPV ensures a lower LOD than CV and it is one of the most sensitive electroanalytical techniques. However, the use of a sophisticated potential wave, with a careful setting of the different parameters, requires an elaborated read out electronics and may not be the best technique to choose for practical real-life applications.

**Figure 4:**
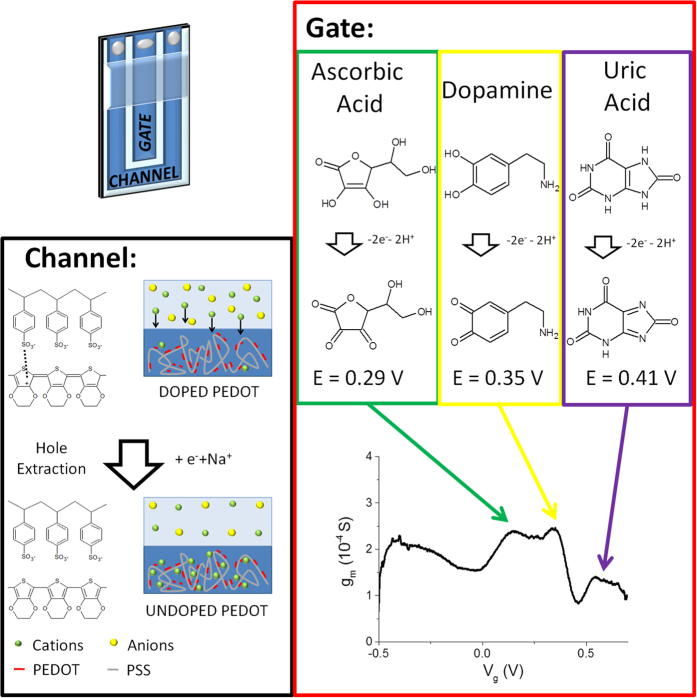
Working principle of the selective all-PEDOT:PSS OECT. The oxidation reactions of AA, DA and UA take place at different electrochemical potentials and, consequently, also at different gate potentials in an OECT. When a faradic process occurs at the gate electrode, more cations are injected in the PEDOT:PSS of the channel leading to an extraction of holes from the conductive polymer. Consequently, the gate effect is enhanced by a rise of the trans-conductance value. The OECT sensor indentifies the contributions of different analytes as peaks in the trans-conductance plot.

Although step potential techniques are widely employed for the selective amperometric detection through the use of sensors based on chemically modified electrodes, this approach has never been applied to OECTs. Here, for the first time, we explore this opportunity by recording the transfer curves of an OECT in solutions containing different analytes at the same time. A transfer curve is a I_d_-V_g_ curve which is acquired by linearly varying the potential applied to the gate electrode and recording the current that flows at the drain collector. Therefore the gate electrode carries out a potential scan that is very similar to that applied to the working electrode in a CV. In the OECT operation mode, the redox process induced by the gate-potential variation affects the electrical conductivity of the channel. The evaluation of the different contributions to the electrical signal due to various analytes is finally made possible by a V_g_ scan, with the key advantage that the transistor configuration of the OECT provides an intrinsic signal amplification (see Fig. 4).

The transfer curve obtained for the blank solution is practically a straight line. This evidence can be explained by considering that, in this condition, the only process occurring at the gate electrode is PEDOT:PSS oxidation. Since PEDOT:PSS exhibits a typical capacitive behavior (see Figure SI2) with a rectangular-shaped CV, also the current that flows at the gate electrode is constant and independent from V_g_ (Figure SI3). Considering that I_g_ represents also the number of charge carriers extracted from the channel, a V_g_ variation exerts a constant action on the channel conductivity independently of the applied V_g_. Therefore, in a solution not containing any analyte the plot I_d_-V_g_ is a straight line.

**Figure 5:**
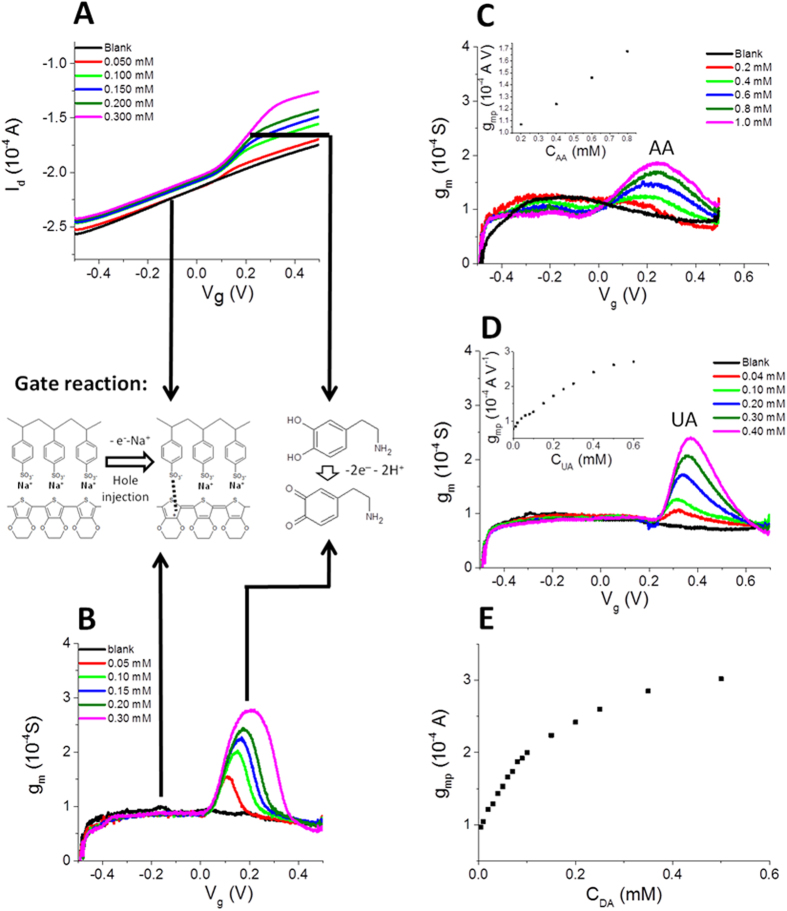
(**A**) Transfer curves recorded in a solutions containing DA at different concentrations. (**B**) Trans-conductance curves obtained from transfer curves reported in Fig. 5A. Trans-conductance curves recorded in a solution containing AA (**C**) and UA (**D**) at different concentrations. In the insets the calibration plots are reported. (**E**) Calibration plot obtained for dopamine. The chemical scheme shows the reactions which take place in different areas of the graphs. Only PEDOT:PSS oxidation occurs in the blank and at low gate voltage (V_g_). In the presence of DA, also the DA oxidation occurs at V_g_ ∼ 0.2 V.

When DA is added to the solution (Fig. 5A), the I_d_-V_g_ curve acquires a sigmoidal-like shape. This behavior can be explained by considering that a new electrochemical process, i.e. dopamine oxidation, takes place at the gate electrode (Fig. 5). This process occurs exclusively at an electrochemical potential that is characteristic of dopamine and it affects the gate current, and consequently I_d_, only at a specific V_g_. Since DA oxidation leads to a I_g_ increase, likewise the gate action increases and more charge carriers are extracted from the channel with respect to the blank solution, leading to a higher I_d_ decrease. Since the redox current is directly proportional to DA concentration, the I_d_ decrement increases by increasing DA concentration.

Actually, our measurements demonstrate that the presence of a redox compound in the electrolyte solution increases the gate action on I_d_. In order to highlight this phenomenon, we also studied the trans-conductance, g_m_. Trans-conductance is the electrical characteristic relating the current through the output of a device to the voltage across the input of a device. In order to calculate the trans-conductance as a function of V_g_, the transfer curve I_d_ (V_g_) was derived according to its definition given in equation 1.


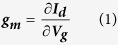


Since in an OECT the gating effect is mediated by the electrolyte solution, the presence of an electro-active compound can induce a significant variation in its trans-conductance when an appropriate V_g_ is applied. In particular, the occurrence of a faradic process associated to the analyte oxidation leads to an increase in current modulation, and hence of the trans-conductance value. Figure 5B reports the trans-conductance curves obtained from the transfer curves recorded in a solution containing DA at different concentrations and Figure SI4 shows the trans-conductance curves obtained in the micromolar range. In the trans-conductance plot the DA signal displays a peak-like shape and the intensity of this peak is linearly related to DA concentrations in the 0.005–0.100 mM range.

The same result can be obtained in solutions containing AA or UA (see Fig. 5D). For each analyte the calibration plots (Fig. 5) were constructed and the characteristic parameters are reported in Table 1. It is worth noting that for all the tested analytes the OECT LOD values are lower than those observed by CV, the electrochemical technique that uses the same shape of the potential wave. Moreover, looking at the sensitivity values normalized with respect to the gate area so that a comparison with DPV results is possible, the sensitivities are comparable or even higher than DPV measurements, highlighting the great advantage due to the signal amplification deriving from the transistor architecture. Our device exhibits a limit of detection for DA equal to 6 μM, which is still higher than those achievable with OECT having a Pt gate electrode^23^,^24^, but comparable to that obtained by more sophisticated techniques such as for example fast scan cyclic voltammetry at a patterned 10 μm gold microelectrode using an additional PEDOT:PSS OECT to amplify the FSCV signal^25^. The strength of our work lies in the demonstration that an all-plastic OECT enables the discrimination among DA and interfering species. Obviously, future work will be devoted to the LOD improvement, for example by changing the gate electrode geometry.

**Figure 6:**
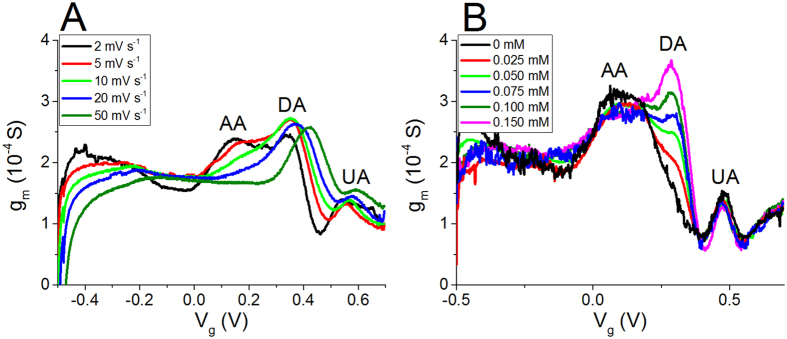
Trans-conductance curves recorded (**A**) in a solution containing AA (1 mM), DA (0.1 mM) and UA (0.5 mM) at different scan rates and (**B**) in a solution containing dopamine at different concentrations plus 0.1 mM uric acid and 0.5 mM ascorbic acid (fixed concentrations) at 0.002 V s^−1^.

Trans-conductance curves were recorded for a solution containing DA, AA and UA simultaneously in order to demonstrate the applicability of this approach to the selective detection of the three analytes at the same time (Figs 4 and 6A). Figure 6A shows that the resolution between the three waves associated to the redox compounds depends on the scan rate as previously observed for CV, being the best for the lowest scan rate. The different redox waves can be assigned to the different redox species considering the peak potentials determined by CV. The peaks at 0.16, 0.34 and 0.54 V are thus ascribed to AA, DA and UA, respectively. These results have been experimentally confirmed by sequentially adding different amounts of each analyte to a solution containing all the compounds, in order to identify which peak increases.

The scan-rate variation affects the OECT response in a way that is similar to that observed in CV experiments with a 3-electrode cell set-up. By increasing the scan rate, the AA peak moves towards higher V_g_ and it merges with DA peak. Such phenomenon can be ascribed to the slow kinetics of AA electro-oxidation at the gate electrode, as previously stated. Nevertheless, the shift of AA peak does not affect the intensity of the DA peak up to the highest tested scan rate. This observation agrees with the literature data on amperometric detection^25^, which reports that it is possible to avoid the AA interference in DA determination by working at high scan rate.

The final part of our work is devoted to demonstrate that the described step potential technique can be successfully employed in the selective DA detection in the presence of AA and UA as interferents. Figure 6B shows the trans-conductance curves recorded at a scan rate of 0.002 V s^−1^ in a solution containing 0.5 mM AA and 0.1 mM UA while increasing DA concentration. The figure clearly shows that DA peak is well separated from AA and UA signals and that the peak current linearly depends on the dopamine concentration, with a slope equal to 1.32 ± 0.07 S M^−1^. Such value is the same as that obtained when a solution containing only DA was studied in the same condition of scan rate (Fig. SI5). This is a further confirmation that all-PEDOT:PSS OECT devices can be effectively used as selective sensors for DA detection when a step potential technique is employed.

In order to gain a better insight into the interfering species effects on the DA response, trans-conductance curves were recorded at a fixed DA concentration while adding UA and AA to the solution. The results of this study are discussed in more detail in Supplementary Information Section (Figure SI6).

## Conclusions

Organic electrochemical transistors have been proposed as chemical sensors thanks to their remarkable features such as signal amplification, very low operating potentials and adsorbed power. Nevertheless, the lack of selectivity hinders their widespread use in real-life applications. Potentiodynamic techniques are commonly employed to separate the redox waves associated to different analytes for common 3-electrode electrochemical sensors based on an amperometric transduction, but this approach had not yet been explored for OECT sensing. We explored a new approach to selectively identify and determine the contributions of different analytes to the OECT electrical output signal through a linear scan of the gate potential. We have used OECTs entirely made of PEDOT:PSS (both conductive channel and gate) in order to take advantage of the peculiar electrochemical properties of the conducting polymer.

By CV and DPV characterizations in a 3-electrode cell set-up, we achieved a deeper insight into the electrochemical processes that occur at the polymer gate electrode, showing that: (i) the oxidation of different analytes occurs at different potentials and (ii) the scan rate affects the separation between the redox waves by influencing the kinetics of charge transfer reactions. We assessed how OECTs can profit from PEDOT:PSS electrochemical features by selectively detecting the electro-oxidation of three different analytes (ascorbic acid, uric acid and dopamine) present in the same solution, as they occur at different gate potentials. The signal related to each one of the three different analytes can be individually detected and resolved by recording the trans-conductance, obtaining a linear response for all the analytes. The all-PEDOT:PSS OECT sensor can thus separate the redox waves associated to each compound and the scan rate is the key parameter to obtain both selectivity and a good resolution.

The results reported here also demonstrate that all-PEDOT:PSS OECTs sensitivities and limits of detection are comparable or even higher than the ones achievable by DPV. In particular, the limits of detection are comparable to those obtained by DPV, a technique that employs a sophisticate potential wave and read-out system in order to maximize the performance of electrochemical sensors and that can hardly be considered a viable readout method in practical applications. The validity of the proposed approach has been assessed by using all-PEDOT:PSS OECTs as sensors for dopamine in presence of ascorbic acid and uric acid. The resulting interference of both compounds was very low, even if the concentrations of the interfering agents were noticeably higher than the dopamine’s one. We were able to reach a limit of detection of 6 μM, which is still higher than the value desirable for DA detection in biological samples, but the results described here open new perspectives on a simple tool that greatly improves the performance of all-PEDOT:PSS OECTs, by demonstrating the proof-of-principle of their selectivity and, thus, the possibility to employ them as bioanalytical sensors in real-life applications.

## Experimental

### OECT fabrication

The OECT was prepared by spin coating CLEVIOS PH 1000 suspension (PEDOT:PSS) on a glass slide at 500 rpm per 3 s. Figure 1A reports a schematic representation of the device; the external PEDOT:PSS stripe was used as the source-drain channel while the inner one worked as the gate electrode.

### Electrochemical Measurements

The electrochemical characterizations were carried out by CV and DPV in a three electrode cell using a 0.1 M phosphate buffer solution (PBS), pH 5.5, as electrolyte. The electrochemical potentials were measured with respect to an aqueous saturated calomel electrode (SCE), a Pt wire was used as the counter electrode and a PEDOT:PSS thin film deposited on a glass slide was used as the working electrode. The electrochemical responses were acquired employing a CH Instrument 660C that was controlled by personal computer CHI Software. For DPV, the instrumental variables were studied and the optimum conditions resulted: pulse amplitude: 0.050 V; increment: 0.004 V; sample width: 4 s; pulse width: 5 s; pulse period: 10 s; sensitivity: 1 × 10^−4^A.

Two Sourcemeters 2400 SMU (KEITHLEY) controlled by a personal computer via a homemade LAB-VIEW software were simultaneously employed in order to carry out the electrical measurements (Fig. 1C) by applying source-drain (V_d_) and source-gate (V_g_) potentials and measuring the respective currents (I_d_, I_g_). A defined area both for the gate (0.3 × 0.3 cm^2^) and the channel (0.3 × 3.0 cm^2^) was left unprotected and exposed to the electrolyte solution. The OECT was dipped in PBS under magnetic stirring and the transfer curves were acquired in absence and in presence of the analytes at different concentrations. The trans-conductance curves were obtained by deriving the transfer curves with origin software. The OECT performance has been evaluated by considering the sensitivity and the limit of detection.

## Additional Information

**How to cite this article**: Gualandi, I. *et al.* Selective detection of dopamine with an all PEDOT:PSS Organic Electrochemical Transistor. *Sci. Rep.* **6**, 35419; doi: 10.1038/srep35419 (2016).

## Supplementary Material

Supplementary Information

## Figures and Tables

**Table 1:** Performance of CV, DPV and OECT in the detection of AA, DA and UA.

	Three electrode cell				OECT	
	CV		DPV			
	E_pa_^a^ (V vs SCE)	Sensitivity^b^(LOD)^c^	Sensitivity^b^(LOD)^c^	Sensitivity^e^	Sensitivity^d^(LOD)^c^	Sensitivity^e^
AA	0.29	0.063 ± 0.005(1 10^−4^)	0.00967 ± 0.0003(2 10^−5^)	0.193 ± 0.003	0.102 ± 0.004(7 10^−5^)	0.85 ± 0.03
DA	0.35	0.109 ± 0.009(3 10^−4^)	0.448 ± 0.006(2 10^−6^)	9.0 ± 0.1	1.09 ± 0.04(6 10^−6^)	9.1 ± 0.3
UA	0.41	0.090 ± 0.002(2 10^−4^)	0.065 ± 0.001(6 10^−6^)	1.30 ± 0.02	0.40 ± 0.01(2 10^−5^)	3.33 ± 0.08

^a^anodic peak potentials evaluated by CV for 1 mM solutions.

^b^expressed in A M^−1^ cm^−2^.

^c^expressed in M.

^d^expressed in S M^−1^.

^e^normalized sensitivity expressed in S M^−1^ cm^−2^. The DPV and OECT sensitivities are normalized to pulse amplitude and gate area, respectively.

## References

[b1] Zen J.-M., Kumar A. S. & Tsai D.-T. Recent Updates of Chemically Modified Electrodes in Analytical Chemistry. Electroanal. 15, 1073–1087 (2003).

[b2] Wring S. A. & Hart J. P. Chemically modified, carbon-based electrodes and their application as electrochemical sensors for the analysis of biologically important compounds. A review. Analyst. 117, 1215–1229 (1992).

[b3] Atta N. F., Galal A., Abu-Attia F. M. & Azab S. M. Carbon Paste Gold Nanoparticles Sensor for the Selective Determination of Dopamine in Buffered Solutions. J. Electrochem. Soc. 157, F116–F123 (2010).

[b4] Spooren A. *et al.* Resistance of the dopamine D4 receptor to agonist-induced internalization and degradation. Cell. Signal. 22, 600–609 (2010). 19932171 10.1016/j.cellsig.2009.11.013

[b5] Shang F. *et al.* Selective nanomolar detection of dopamine using a boron-doped diamond electrode modified with an electropolymerized sulfobutylether-β- cyclodextrin-doped poly(N-acetyltyramine) and polypyrrole composite film. Anal. Chem. 81, 4089–4098 (2009). 19382752 10.1021/ac900368m

[b6] Sun C. L., Lee H.-H., Yang J.-M. & Wu C.-C. The simultaneous electrochemical detection of ascorbic acid, dopamine, and uric acid using graphene/size-selected Pt nanocomposites. Biosens. Bioelectron. 26, 3450–3455 (2011). 21324669 10.1016/j.bios.2011.01.023

[b7] Teymourian H., Salimi A. & Khezrian S. Fe_3_O_4_ magnetic nanoparticles/reduced graphene oxide nanosheets as a novel electrochemical and bioeletrochemical sensing platform, Biosens. Bioelectron. 49, 1–8 (2013). 23708810 10.1016/j.bios.2013.04.034

[b8] Lian Q. *et al.* Simultaneous determination of ascorbic acid, dopamine and uric acid based on tryptophan functionalized grapheme, Anal. Chim. Acta 823, 32–39 (2014). 24746351 10.1016/j.aca.2014.03.032

[b9] Zanardi C., Terzi F. & Seeber R. Polythiophenes and polythiophene-based composites in amperometric sensing. Anal. Bioanal. Chem. 405, 509–531 (2013). 22941065 10.1007/s00216-012-6318-7

[b10] Lin P. & Yan F. Organic Thin-Film Transistors for Chemical and Biological Sensing. Adv. Mater. 24, 34–51 (2012). 22102447 10.1002/adma.201103334

[b11] Cramer T. *et al.* Water-gated organic field effect transistors – opportunities for biochemical sensing and extracellular signal transduction. J. Mater. Chem. B. 1, 3728–3741 (2013). 10.1039/c3tb20340a 32261126

[b12] Malinauskas A. Electrocatalysis at conducting polymers. Synth. Met. 107, 75–83 (1999).

[b13] Dietrich M., Heinze J., Heywang G. & Jonas F. Electrochemical and spectroscopic characterization of polyalkylenedioxythiophenes. J. Electroanal. Chem. 369, 87–92 (1994).

[b14] Li G. & Pickup P. G. Ion transport in poly(3,4-ethylenedioxythiophene)–poly(styrene-4-sulfonate) composites. Phys. Chem. Chem. Phys. 2, 1255–1260 (2000).

[b15] Bobacka J., Lewenstam A. & Ivaska A. Equilibrium potential of potentiometric ion sensors under steady-state current by using current-reversal chronopotentiometry. J. Electroanal. Chem. 509, 27–30 (2001).

[b16] Cui X. & Martin D. C. Electrochemical Deposition and Characterization of Poly (3,4-Ethylenedioxythiophene) on Neural Microelectrode Arrays. Sens. Actuat B-Chem. 89, 92–102 (2003).

[b17] Lisowska-Oleksiak A. & Kupniewska A. Transport of alkali metal cations in poly (3,4-ethylenethiophene) films. Solid State Ionics. 157, 241–248 (2003).

[b18] Han D., Yang G., Song J. Niu L. & Ivaska A. Morphology of electrodeposited poly(3,4-ethylenedioxythiophene)/poly(4-styrene sulfonate) films. J. Electroanal. Chem. 602, 24–28 (2007).

[b19] Basiricò L., Cosseddu P., Fraboni B. & Bonfiglio A. Inkjet printing of transparent, flexible, organic transistors. Thin Solid Films 520, 1291–1294 (2011).

[b20] Basiricò L. *et al.* Electrical characteristics of ink-jet printed, all-polymer electrochemical transistors. Organic Electroics. 13, 244–248 (2012).

[b21] Demelas M. & Scavetta E., Basiricò, L., Rogani, R. & Bonfiglio, A. A deeper insight into the operation regime of all-polymeric electrochemical transistors. Appl. Phys. Lett. 102, 193301 (2013).

[b22] Gualandi I. *et al.* A simple all-PEDOT:PSS electrochemical transistor for ascorbic acid sensing. J. Mater. Chem. B. 3, 6753–6762 (2015). 10.1039/c5tb00916b 32262468

[b23] Tang H., Lin P., Chan H. L. W. & Yan F. Highly sensitive dopamine biosensors based on organic electrochemical transistors. Biosens. Bioelectron. 26, 4559–4563 (2011). 21652201 10.1016/j.bios.2011.05.025

[b24] Liao C., Zhang M., Niu L., Zheng Z. & Yan F. Organic electrochemical transistors with graphene-modified gate electrodes for highly sensitive and selective dopamine sensors. J. Mater. Chem. B. 2, 191–200 (2014). 10.1039/c3tb21079k 32261606

[b25] Tybrandt K., Kollipara S. B. & Berggren M. Organic electrochemical transistors for signal amplification in fast scan cyclic voltammetry. Sensor. Actuat. B-Chem. 195, 651–656 (2014).

