# Supplementary material for: Selective detection of dopamine with an all PEDOT:PSS Organic Electrochemical Transistor
Source: Sci Rep. 2016 Oct 14;6:35419. doi: 10.1038/srep35419 (PMC5064404; doi:10.1038/srep35419)
Supplement: Supplementary Information [file srep35419-s1.pdf]

Supplementary Materials

Selective detection of dopamine with an all PEDOT:PSS Organic Electrochemical Transistor

Isacco Gualandi[1], Domenica Tonelli[1], Federica Mariani[1], Erika Scavetta[1*], Marco Marzocchi[2], Beatrice Fraboni[2]

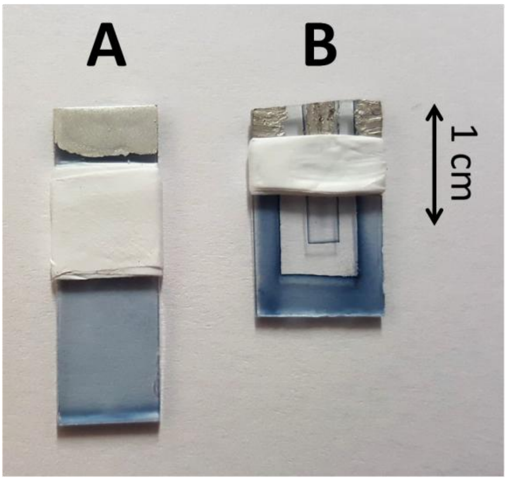

Fig. SI 1 Image of the electrochemical (A) and OECT (B) sensors.

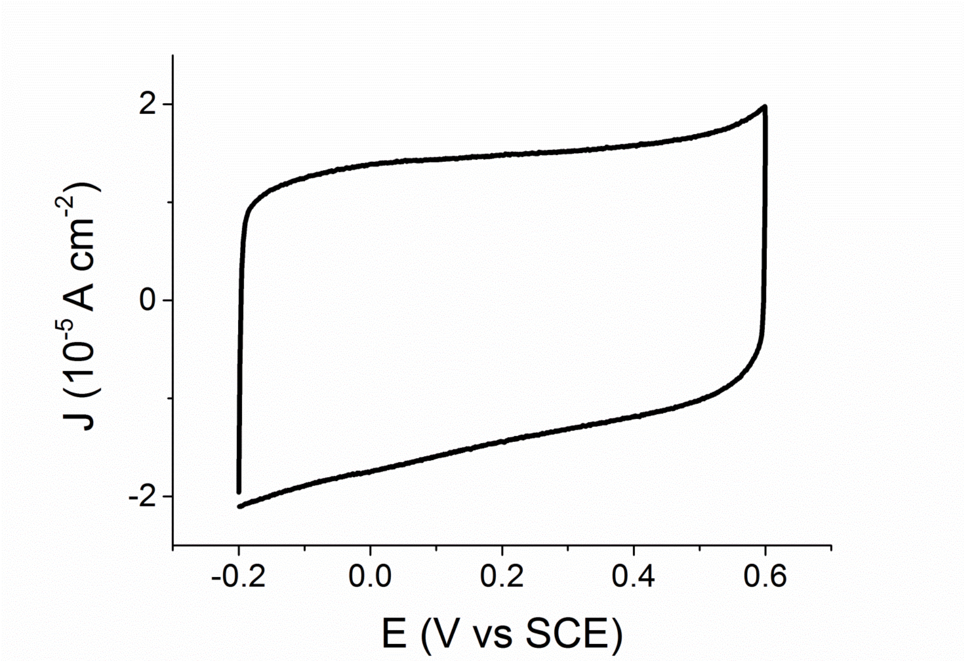

Fig SI. 2 CV recorded in 0.1 M PBS, pH 5.5

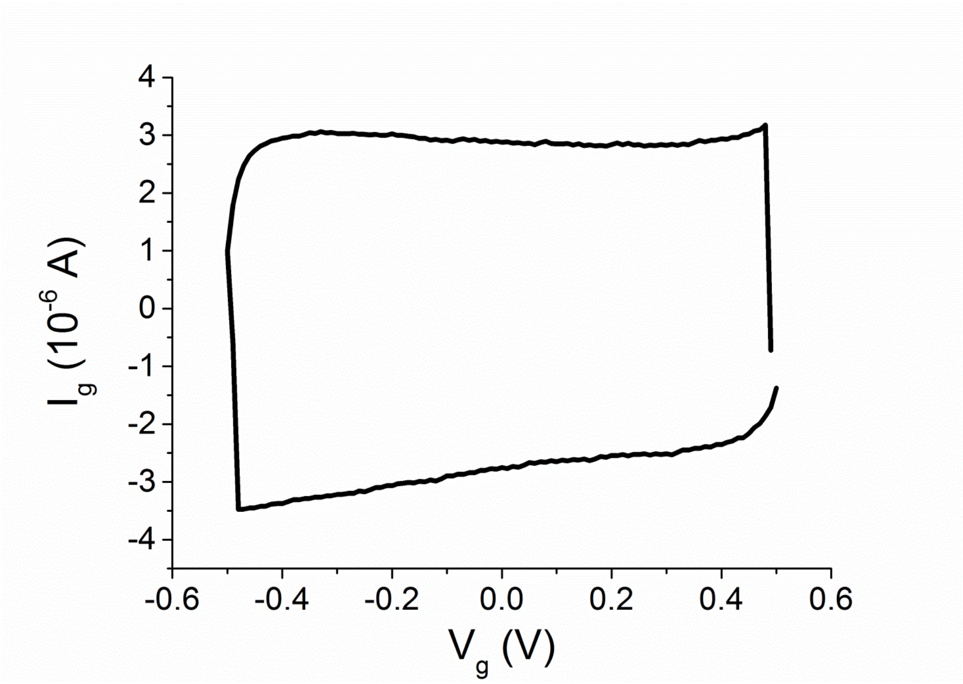

Fig SI. 3 I_g-V_g curve recorded in 0.1 M PBS, pH 5.5 . Also the return was record

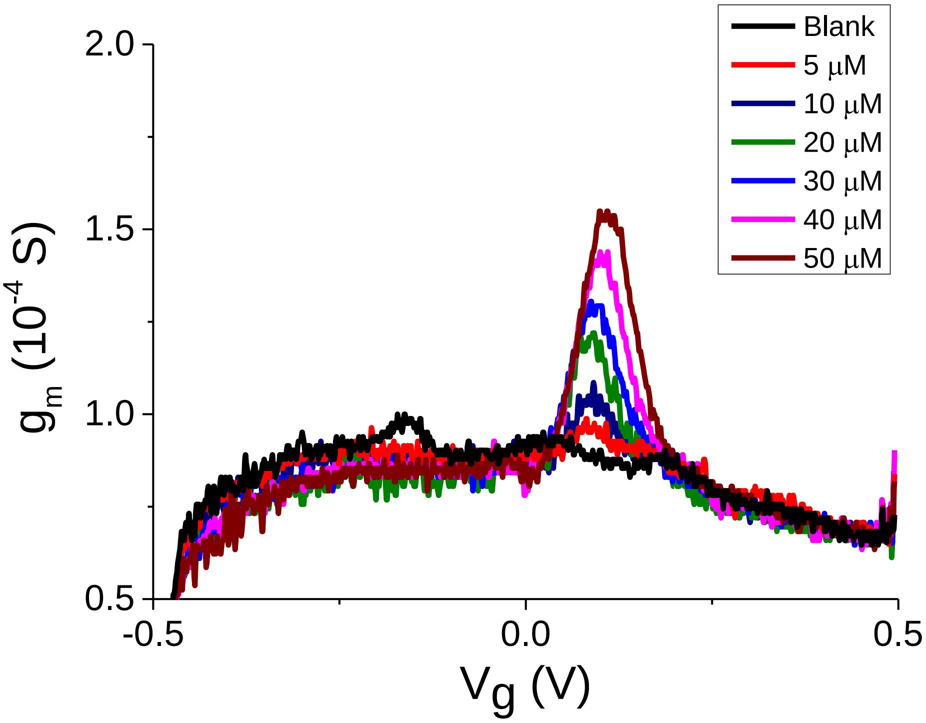

Fig. SI 4 Response of the sensor in micro molar range

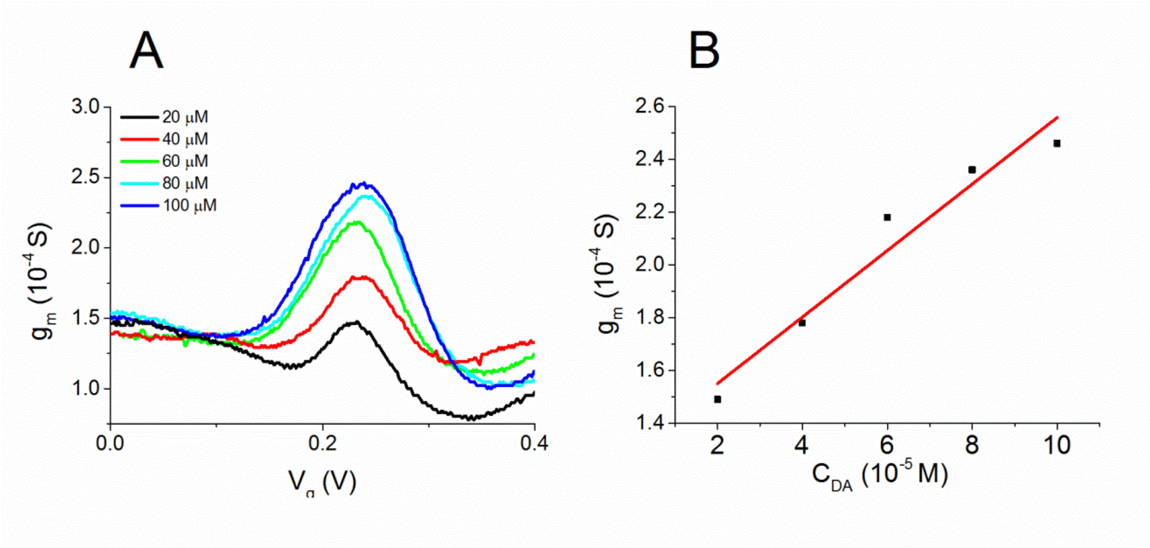

Fig. SI 5. A) Trans-conductance plot obtained for DA for a scan rate equal to 0.002 V s^{-1}. B) Trans-conductance peak values vs dopamine concentration.

Interfering study

In order to gain a better insight into the interfering species effects on the DA response, trans-conductance curves were recorded at a fixed DA concentration while adding UA and AA to the solution.

In order to minimize the AA and UA contribution to the signal, a high scan rate (0.050 V s^{-1}) was employed. In such a condition, Figure SI 14 A shows the trans-conductance curves recorded in a solution of 0.1 mM DA while UA concentration was increased from 0.1 mM to 6.4 mM. The increase of UA concentration leads to a decrease of DA response probably because UA competes for the occupancy of PEDOT redox sites, causing a saturation of the sensor response. However the DA signal decreases of only 20 % when UA concentration which is 64 times higher than DA concentration.

Figure SI 14 B shows the trans-conductance curves recorded in a solution of 0.1 mM DA while the concentration of AA was increased from 0.1 mM to 0.4 mM. Also in this case the addition of AA produces a decrease of DA signal. The addition of 0.4 mM AA leads to a reduction of dopamine signal of about 20 %. The extent of AA interference is higher than that exerted by UA, probably due to the lower AA oxidation potential.

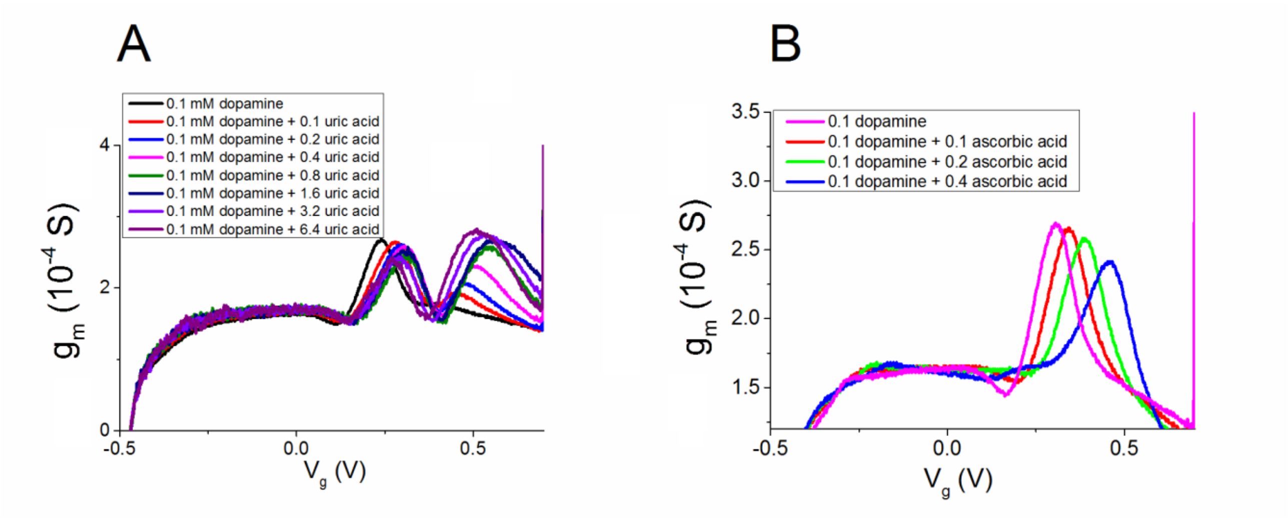

Fig SI. 6 Trans-conductance curves obtained in a solution containing 0.1 mM DA plus UA (A) and AA (B) at different concentrations.
